# The use of Botulinum Toxin Type A in patients in out-of-hospital intensive care in Germany—results from a multidisciplinary online survey

**DOI:** 10.3389/fneur.2025.1639242

**Published:** 2025-11-21

**Authors:** Victoria Ohla, Martin Groß

**Affiliations:** 1 Deutsche Interdisziplinäre Gesellschaft für Außerklinische Beatmung und Intensivversorgung (DIGAB) e.V., Göttingen, Germany; 2 MEDIAN Klinik Bad Tennstedt, Bad Tennstedt, Germany; 3 Department of Health Services Research, Faculty of Medicine and Health Sciences, Carl von Ossietzky Universitat Oldenburg, Oldenburg, Germany

**Keywords:** Botulinum Toxin Type A, out-of-hospital intensive care, spasticity, sialorrhea, hypersalivation, aspiration, BoNT-A

## Abstract

**Introduction:**

In Germany, approximately 23.000 patients live in of out-of-hospital intensive care settings. About 80% suffer from neurological diseases and are prone to spasticity and sialorrhea, both of which can be effectively treated with Botulinum Toxin Type A (BoNT-A). This study investigates the utilization, barriers, and training needs associated with BoNT-A application.

**Methods:**

An online questionnaire with 10 questions was developed by the German Interdisciplinary Society for Out-of-Hospital Ventilation and Intensive Care (DIGAB). The survey was distributed via the mailing lists of the DIGAB, the German Society for Respiratory Therapy, and the nursing supervisors of the Deutsche Fachpflege nursing service, as well as to speech and language therapists through a private mailing list.

**Results:**

The survey was sent to 702 recipients, with 41 healthcare professionals completing the survey. Only 160 (20%) of 789 patients with spasticity and 111 (14%) of 816 patients with sialorrhea or salivary aspiration received BoNT-A treatment. Barriers included the lack of trained providers, logistical challenges, and uncertainty regarding cost coverage. Participants emphasized the importance of staff training, availability of medical specialists and treatment in the home environment.

**Conclusion:**

Despite its potential to facilitate care, to improve quality of life, and to promote participation in out-of-hospital intensive care, BoNT-A remains underused. Key requirements include education on multidisciplinary treatment of spasticity and sialorrhea, adequate reimbursement for BoNT-A administration, a coordinating role for the neurologist in the regulatory framework of out-of-hospital intensive care, and integration of BoNT-A in treatment guidelines are required. Further research should collect patient-level data on spasticity, dystonia, sialorrhea, and BoNT-A treatment, and examine healthcare delivery across different healthcare structures.

## Introduction

Severe spasticity, sialorrhea, and related complications impair patients’ participation and their quality of life (1–3). Treatment of spasticity should be provided by a multidisciplinary team, supervised by a specialist in physical medicine and rehabilitation or in neurology with a subspecialisation in rehabilitation (4). Multidisciplinary treatment of sialorrhea is considered to achieve optimal benefits for patients as well (5).

Botulinum toxin A (BoNT-A) is a neurotoxin that selectively inhibits the presynaptic release of acetylcholine from the terminal of the second motoneuron at the neuromuscular junction, thereby reducing muscle tone after intramuscular administration (6). The dosage depends on the affected muscles, the severity of spasticity, and the duration of the effect (see Table 1 for an overview of the maximum dosages of the different types of BoNT-A used for adult spasticity). Patients from Europe and the US reported a mean frequency of 3.6 injections per year (7). When injected into the salivary glands its anticholinergic effect leads to a reduced production of saliva (8). The injection of incobotulinumtoxin A—30 units into each parotid gland and 20 units into each submandibular gland in adults—must be repeated every 16 weeks.

**Table 1:** Maximum dosage of BoNT-A in adult spasticity, U, units^*^.

Body region	Abobobotulinumtoxin A	Onabobotulinumtoxin A	Incobobotulinumtoxin A
Upper extremity	1,500 U	240 U	500 U
Shoulder only	500 U	Not approved in Germany	250 U
Lower extremity	1,500 U	400 U	Not approved in Germany
Total	1,500 U	400 U	–

* Units are not comparable across BoNT-A formulations, and approval regarding underlying conditions differs substantially between products.

The effectiveness of BoNT-A in treating spasticity and sialorrhea in children and adults has been demonstrated in randomized placebo-controlled trials (9–12). BoNT-A has the potential to reduce pain, improve patient comfort, facilitate daily care routines, and enhance long-term care and rehabilitation outcomes, and it may help to reduce aspiration pneumonia in cases of salivary aspiration (13). A multimodal approach and adjunctive therapies are both used to enhance the treatment effect of BoNT-A in spasticity (14). BoNT-A is also regarded as a first-line treatment for cranial or cervical dystonia (15). Neutralizing antibodies may reduce the efficacy and lead to secondary treatment failure, and they are reported in patients treated with abobotolinumtoxin A and onabotulinumtoxin A, but not in patients treated only with incobotulinumtoxin A (16). The most relevant side effects of BoNT-A in patients receiving out-of-hospital intensive care are dysphagia and respiratory symptoms (17, 18). Although high doses carry a risk of iatrogenic botulism (19), the safe administration of doses up to 1,200 MU has been demonstrated for incobotulinumtoxin A, supporting its use in severe, widespread spasticity in out-of-hospital intensive care (20).

Out-of-hospital intensive care is a specialized service for patients with severe, chronic conditions requiring continuous nursing support, such as tracheostomy management or mechanical ventilation. In this context, qualified nurses provide round-the-clock monitoring and interventions such as endotracheal suctioning. At home, one-to-one nursing is required; in facilities, ratios are typically closer to one-to-three. Beyond medical stabilization, the focus lies on autonomy, quality of life, and social participation (21).

In Germany, approximately 23,000 patients currently live in out-of-hospital intensive care settings (22). In a large sample of 2,040 patients in out-of-hospital intensive care, 50% suffered from brain disease, 24% from neuromuscular disease, and 5% from spinal cord injury. In 16% the underlying disease was pulmonary, and in 5% another underlying disease was present. Eighty percent of all patients suffered from dysphagia, and 69% had a percutaneous endoscopic gastrostomy tube. Eighty percent had a tracheal tube, 25% needed invasive ventilation, and 7% had noninvasive ventilation. An unresponsive wakefulness syndrome was present in 18% of all patients (23).

Such patients are predisposed to spasticity, dysphagia, sialorrhea and salivary aspiration. However, the complex care demands, reduced mobility, and decentralized health services pose major challenges for healthcare providers and caregivers. German regulations mandate biannual assessments of the patients’ potential to be weaned from mechanical ventilation or to have a tracheal cannula removed—even in progressive disorders such as genetic neuromuscular diseases or amyotrophic lateral sclerosis. These assessments require specific physician qualifications: pulmonologists are eligible, whereas neurologists must meet additional requirements. Since the regulation’s introduction on March 3, 2022, a shortage of qualified physicians has led to significant delays (24). To mitigate this gap, some large nursing services have introduced supervisory nursing roles, though delegation from a physician remains necessary (25, 26).

Since 2024, the German Interdisciplinary Society for Out-of-Hospital Ventilation and Intensive Care (DIGAB) e. V. has been formally committed, according to its statutes, to the “support and promotion of care and care structures for individuals requiring out-of-hospital ventilation or intensive care.” DIGAB is the only interdisciplinary society in Germany dedicated to this field, comprising nurses, physicians, therapists, patients, family caregivers, and industry representatives. Founded in 1997 as the “Working Group for Home Ventilation and Ventilator Weaning e. V.”, and renamed in 2010 as the “German Interdisciplinary Society for Out-of-Hospital Ventilation (DIGAB) e. V.,” the organization has gradually expanded its scope from home ventilation to the entire spectrum of out-of-hospital intensive care. DIGAB has been actively engaged in scientific work and has contributed to eight national guidelines on mechanical ventilation and out-of-hospital intensive care so far.

Despite its clinical importance, the utilization of BoNT-A in out-of-hospital intensive care—as well as the structural and systemic barriers inherent to this specialized setting—remain understudied. This survey aims to assess the extent and limitations of BoNT-A use in this context, and to outline strategies for improving care quality and sustainability.

## Methods

The survey was conducted under the auspices of the DIGAB. The topics of the questionnaire were derived from a literature review and subsequently endorsed by the DIGAB’s board and its multidisciplinary section for neurology and neurorehabilitation. The questionnaire consisted of nine closed questions and one open question and addressed the following topics:

- Professional background and workplace of the participants.
- The number of patients with spasticity and chronic sialorrhea or salivary aspiration cared for by participants in the past 6 months, and the proportion treated with BoNT-A.
- Barriers to BoNT-A utilization.
- Training needs.
- Suggestions for improving patient care (open-text response).

The topics of spasticity and chronic sialorrhea/salivary aspiration were illustrated using representative clinical images.

Data collection was carried out using the digital survey platform SoSci Survey in April 2024. The survey was distributed to 637 DIGAB members, 34 members of the German Society for Respiratory Therapy (DGA), 25 specialized nursing supervisors from Deutsche Fachpflege Holding GmbH, and six speech and language therapists via a private mailing list, as speech and language therapists were underrepresented in all other mailing lists. All recipients received the same survey link.

Only responses from healthcare professionals who completed the questionnaire in full were included in the analysis. Data from respondents who answered “do not know” to three or more of the five questions regarding the number of patients with spasticity or sialorrhea or the number of patients who had received BoNT-A because of those conditions were excluded. These criteria were used to address careless responding (27).

Quantitative data from closed questions were analyzed descriptively; frequencies are reported as counts and percentages. For continuous variables, medians and interquartile ranges were calculated. Open-text responses were evaluated using qualitative content analysis.

## Results

The survey was distributed to 702 recipients of whom 41 met the inclusion criteria. Table 2 presents an overview of the respondents’ characteristics. Most of the respondents were nurses (*n* = 30, 73%), followed by respiratory therapists (*n* = 10, 24%), doctors from different specialties (*n* = 9, 22%) and speech and language therapists (*n* = 4, 10%). Because some participants held multiple qualifications (e.g., a nurse additionally certified as a respiratory therapist), percentages exceeded 100%. The respondents predominantly worked in an out-of-hospital intensive care setting: shared living communities or patients’ homes (*n* = 29, 71%), nursing homes (*n* = 7, 17%) or nursing homes specialized in neurorehabilitation (*n* = 2, 5%). Of 789 reported patients with spasticity, 160 (20%) were treated with BoNT-A. Among 816 reported patients with chronic sialorrhea or salivary aspiration, 111 (14%) received BoNT-A. Table 3 summarizes the number of patients with spasticity and chronic sialorrhea/salivary aspiration who were treated or cared for by the participants within the past 6 months, as well as the proportion of these patients who received BoNT-A. The median number of patients reported to be treated with BoNT-A per respondent was 0.5 for spasticity and 0 for sialorrhea.

**Table 2:** Characteristics of respondents.

Occupational groups^*^	*n* (%)
Specialized nursing (adults)	24 (59%)
Specialized nursing (children)	5 (12%)
Specialized nursing (elderly)	1 (2%)
Respiratory therapy	10 (24%)
Speech and language therapy	4 (10%)
Physiotherapy	0 (0%)
Occupational therapy	0 (0%)
Pulmonology	3 (7%)
Neurology	3 (7%)
Anesthesiology	2 (5%)
Pediatrics	0 (0%)
General practitioners	0 (0%)
Other medical specialists	1 (2%)
Other professions	2 (5%)

Work locations^*^	*n* (%)
Out-of-hospital intensive care (living community or patient’s home)	29 (71%)
Out-of-hospital intensive care (nursing home)	7 (17%)
Out-of-hospital intensive care (nursing home with specialization in neurorehabilitation, “Phase F”)	2 (5%)
Acute hospital (incl. outpatient clinics)	5 (12%)
Weaning center	4 (10%)
Respiratory (early) rehabilitation	2 (5%)
Sleep laboratory	1 (2%)
Spinal cord injury center	0 (0%)
Neurological rehabilitation facility	2 (5%)
Medical practice	3 (7%)
Social-pediatric center	1 (2%)
Medical center for adults with disabilities	1 (2%)
Speech and language therapy practice	1 (2%)
Providers (e.g., medical supply stores)	1 (2%)
Other workplaces	1 (2%)

^*^Multiple answers possible.

**Table 3:** Number of patients in out-of-hospital intensive care treated or cared for by the respondents in the last 6 months before the survey.

Symptom	Total (median, IQR)	Treated with Botulinum Toxin A (median, IQR)
Spasticity	8 (IQR 3.5–25)	0.5 (IQR 0–2)
Chronic sialorrhea/or salivary aspiration	9 (IQR 3.75–20.5)	0 (IQR 0–2)
Spasticity with chronic sialorrhea	5 (IQR 3–20)	Not recorded

Perceived barriers to the use of BoNT-A are detailed in Table 4. The barriers reported most often were the lack of a trained doctor (*n* = 26, 63%), high logistical effort (*n* = 14, 34%) and uncertainty about cost coverage (*n* = 14, 34%). A total of 26 participants (63%) expressed a need for further training in the diagnosis and treatment of spasticity, and 30 participants (73%) indicated a training need for managing chronic sialorrhea/salivary aspiration. Conversely, eight participants (20%) indicated no training needs.

**Table 4:** Reasons for not using Botulinum Toxin A.

Reason^*^	*n* (%)
No provider available	26 (63%)
High logistical effort	14 (34%)
Uncertainty about cost coverage	14 (34%)
Sufficient systemic medication	7 (17%)
Therapy not previously known	6 (15%)
Relatives/caregivers refused therapy	6 (15%)
No perceived benefit from therapy	3 (7%)
Patient refusal	2 (5%)
Other reasons	5 (12%)
Cannot or do not want to assess (opt-out option)	3 (7%)

* Multiple answers possible.

The open question on improving patient care was answered by 12 participants. Their suggestions included:

- Enhanced training and education (*n* = 4), e.g., *“Often lack of knowledge results in non-use of therapies.”*
- Improved access to specialized medical expertise (*n* = 2), e.g., *“In many cases patients are not cared for by a medical specialist.”*
- Preference for local treatment instead of hospitalization (*n* = 2), e.g., *“The patient had to be transported to the hospital to receive his treatment.”*
- Closer collaboration between physicians and therapists (*n* = 1)
- More attentive patient observation (*n* = 1)
- Comprehensive documentation including complementary nursing interventions (*n* = 1)

Additionally, one respondent highlighted the importance of fiberoptic endoscopic evaluation of swallowing (FEES) for tracheostomy management. He also emphasized non-pharmacological interventions, such as swallowing training, cuff deflation techniques and tracheostomy downsizing.

## Discussion

This study highlights the current state of Botulinum Toxin A (BoNT-A) use in the out-of-hospital intensive care setting, identifies barriers to its implementation, and captures educational needs and potential strategies to improve care. Despite BoNT-A being an evidence-based treatment for managing spasticity and chronic sialorrhea—conditions that significantly impact patients’ quality of life—its application remains limited. In this survey, only 20% of patients with spasticity and 14% of those with chronic sialorrhea were reported to have received BoNT-A treatment. These findings align with previous studies that have documented a gap between clinical evidence and real-world practice in Germany regarding spasticity: Less than 10% of patients with spasticity in Germany receive BoNT-A injections, and only 4% of patients with spasticity in nursing homes (28). Given that barriers to BoNT-A treatment are typically higher in out-of-hospital intensive care compared to nursing homes, our study likely overestimated the actual proportion of patients treated.

Healthcare delivery serves as an important indicator of overall system performance. The decentralized structure of the German healthcare system may lead to regional disparities in the provision of BoNT-A, with subsequent variability in treatment accessibility (29). Given the vulnerability of patients requiring out-of-hospital intensive care, it is essential to provide BoNT-A therapy in the patient’s home environment. Transporting tracheostomized or mechanically ventilated individuals poses logistical and medical risks and may lead to significant complications (30). Delivering treatment within the patient’s home environment, rather than in hospital settings, enhances participation, quality of life and patient safety.

The main barriers to BoNT-A use reported in this survey were lack of trained physicians, uncertainty about cost coverage, and high logistical effort. Two of these barriers—lack of trained physicians and uncertainty about reimbursement—have already been addressed by a recent expert consensus article on spastic movement disorders in Germany (28). Comparable literature on sialorrhea, to the best of our knowledge, is not available. High logistical effort, on the other hand, is closely linked to disease severity and is therefore particularly relevant in out-of-hospital intensive care. Additional barriers, such as lack of awareness of therapy with BoNT-A or reliance on systemic antispastic medication, point to a need for targeted educational measures.

Accordingly, a total of 26 respondents (63%) expressed a need for further training in the diagnosis and treatment of spasticity, and 30 participants (73%) indicated a training need for managing chronic sialorrhea/salivary aspiration. Answers to the open question about improving patient care further highlighted the importance of training and education, access to medical specialists, and treatment at home vs. treatment in the hospital. Notably, there is no direct reimbursement for treatment with BoNT-A for spasticity and sialorrhea in Germany (28).

Reimbursement for BoNT-A injections should thus be established. It should cover home visits and portable ultrasound devices, which are often required for safe and effective administration of BoNT-A. Structured educational programs should be established to address knowledge gaps, strengthen clinical competence, and promote the confident and appropriate use of BoNT-A for both spasticity and sialorrhea management (31). Furthermore, multidisciplinary collaboration among physicians, therapists, and nursing staff is essential for the effective treatment of spasticity and sialorrhea (8, 28). Educational initiatives should be tailored to multidisciplinary team settings. In addition to BoNT-A therapy, non-pharmacological interventions remain integral components of patient care and should be integrated into educational programs (see Table 5 for suggested topics).

**Table 5:** Suggested topics for education on diagnosis and treatment of spasticity and sialorrhea.

Spasticity	Sialorrhea
Motor function and movement disorders	Neurology of swallowing, coughing and breathing
Pathways for diagnosis and treatment	Pathways for diagnosis and treatment
Screening for spasticity	Screening for sialorrhea and salivary aspiration
Standardized examination and validated scales	Clinical swallowing examination and airway care score
Clinical practice guidelines	Clinical practice guidelines
Multidisciplinary treatment approach	Flexible endoscopic evaluation of swallowing (FEES)
Physiotherapy	Dysphagia therapy
Botulinum neurotoxin A	Botulinum neurotoxin A
Systemic antispastic medications	Systemic anticholinergic medications
Orthoses	Management of tracheal tubes
Surgical therapies	Radiation of the salivary glands

Furthermore, the current regulations for the biannual medical controls in patients in out-of-hospital intensive care do not address the needs of patients with underlying neurological conditions who form the vast majority of the patients. Severely affected neurological patients require multidisciplinary treatment coordinated by specialized neurologists (32). Therefore, neurologists should be entitled to perform all medical diagnostics, treatments, coordinative tasks and prescriptions described in the regulation for out-of-hospital intensive care (24). The role of the ENT specialist in out-of-hospital intensive care shoud also be strengthened as he may provide not only injections of BoNT-A in the salivary glands but also evaluation of dysphagia and management of tracheal tubes.

### Limitations

The relatively low response rate (41 out of 702 potential participants) among all recipients of the invitation to take part in the survey may limit the generalizability of the findings but a low response rate does not necessarily imply nonresponse bias (33, 34). Low response rates by DIGAB members were found in two other surveys regarding health services for patients in out-of-hospital intensive care and management of tracheobronchial secretions (22, 35). Notably, of 637 DIGAB members, 113 were institutional and 524 were individual members. Individual members not only comprise healthcare professionals but also patients, family caregivers, medical technicians and sales personnel, yet the society’s records do not specify their respective proportions. The response rate among those members meeting the inclusion criteria is thus higher than the above estimate. No data to analyze non-respondent’s characteristics were obtained. The low response rate may reflect the diverse clinical interests of the members representing different professions and work environments, and the DIGAB’s historical focus on out-of-hospital ventilation described in the introduction. While the lack of general practitioners and physiotherapists in the survey may limit the generalizability of the findings, the respondents represent those professions typically working in out-of-hospital intensive care, and most of them reported out-of-hospital intensive care as their workplace accordingly. The fact that the dataset did not include information on the federal or rural vs. urban areas limits its representativeness and hampers identifying determinants of healthcare delivery. Another limitation is the fact, that patient data relied on participant recall rather than systematic documentation. Recall bias could have led to over- or underestimation of the number of patients who had spasticity or sialorrhea or who have been treated with BoNT-A (36). The current approach had been adapted to address several feasibility issues: complex data protection regulations, the regionalized system of medical ethics committees in Germany, the fragmented organization of out-of-hospital intensive care, and most patients not being capable of providing informed consent.

## Conclusion

Despite the well-documented efficacy of Botulinum Toxin A (BoNT-A) in managing spasticity and chronic sialorrhea/salivary aspiration, its use in out-of-hospital intensive care in Germany remains limited. Underutilization of BoNT-A in out-of-hospital intensive care reflects systemic challenges, including limited access to trained medical specialists, insufficient reimbursement, and logistical hurdles. Educational initiatives should be implemented across all disciplines involved in patient care, integrating both pharmacological and non-pharmacological interventions. Additionally, adequate reimbursement mechanisms for BoNT-A application should be established. The role of the neurologist in the regulations for out-of-hospital intensive care should be strengthened, and these regulations should more explicitly reflect the patients' needs. The signficance of BoNT-A should be clarified in treatment guidelines regarding out-of-hospital intensive care for patients with neurological diseases (37). Finally, multidisciplinary outpatient care structures, such as medical centers for adults with disabilities, should be further expanded (38).

Future research should collect individual-level data on spasticity, sialorrhea, dystonia, and BoNT-A treatment, using standardized examinations and validated scales to assess spasticity as well as drooling and airway care (39–42). Additionally, long-term follow-up studies are needed to evaluate the therapeutic effects of BoNT-A, considering outcomes such as contracture development and pneumonia incidence. Furthermore, intersectoral health services research is needed to understand the provision of BoNT-A in upstream health services and to identify regions and characterize healthcare structures with adequate healthcare delivery to severely affected patients in out-of-hospital intensive care. Finally, studies should assess the effectiveness of targeted interventions designed to improve healthcare delivery with respect to BoNT-A therapy in this specialized setting.

## Data Availability

The raw data supporting the conclusions of this article will be made available by the authors without undue reservation.
